# Neck circumference as a metabolic health marker among women with polycystic ovary syndrome (PCOS): a systematic review and meta-analysis

**DOI:** 10.1038/s41366-025-01753-1

**Published:** 2025-04-21

**Authors:** Suvi M. Haapakangas, Noona V. S. Koskenkari, Elisa L. Hurskainen, Riikka K. Arffman, Meri-Maija Ollila, Terhi T. Piltonen

**Affiliations:** Department of Obstetrics and Gynaecology, Medical Research Center Oulu, Research Unit of Clinical Medicine, University of Oulu and Oulu University Hospital, Oulu, Finland

**Keywords:** Metabolic syndrome, Risk factors, Obesity, Pre-diabetes

## Abstract

**Background:**

Polycystic ovary syndrome (PCOS) is associated with obesity, insulin resistance (IR), metabolic syndrome (MetS), and abnormal fat distribution, especially fat excess in the upper body. Neck circumference (NC) reflects the adiposity accumulation in the upper body and may be a valuable and simple screening tool for metabolic risk among women with PCOS.

**Methods:**

A systematic review was conducted using PubMed/Medline and Scopus based on the search terms “neck circumference” and “PCOS”. Studies that examined associations of NC and anthropometric measurements, blood pressure (BP), lipid values, glucose metabolism, MetS, IR, or related disorders among women with PCOS were included. A meta-analysis was performed to compare NC values between women with PCOS and non-PCOS controls.

**Results:**

Of 139 publications, 13 full texts that met the selection criteria were included in the systematic review. Eight studies had non-PCOS controls and were thus eligible for the meta-analysis. Women with PCOS had significantly larger NC compared to non-PCOS controls (SMD: 0.78, 95% CI: 0.31–1.25, *p* = 0.0012). We found a positive association between larger NC and higher waist circumference, hip circumference, triglycerides, systolic BP, fasting insulin, and HOMA-IR or lower HOMA%S and higher prevalence of MetS or IR in the majority of the included studies. Neck circumference cut-off values for MetS varied from 33 cm to 34.25 cm and for IR 34.25 cm up to 42 cm among women with PCOS. Most of the studies were done with Asian populations thus limiting applicability of the study results to other ethnicities.

**Conclusions:**

This meta-analysis demonstrated increased NC among women with PCOS compared to healthy controls. Women with PCOS and larger NC were more insulin resistant and had more MetS-related abnormalities when compared to women with smaller NC with or without PCOS. Data considering NC cut-off values for MetS and IR among women with PCOS are scarce, and further studies are needed, particularly among more varied ethnic populations.

## Introduction

Polycystic ovary syndrome (PCOS) is the most common endocrine disorder [1, 2]. It affects one out of eight women and features polycystic ovarian morphology (PCOM), menstrual dysfunctions and hyperandrogenism [1, 2]. The syndrome is also closely associated with variety of metabolic abnormalities including insulin resistance (IR) [3], obesity [4], abnormal glucose tolerance [5], prediabetes [6], type 2 diabetes (DMT2) [7], cardiovascular risk factors and related diseases [8], and metabolic syndrome (MetS) [9]. Diagnosis of MetS is based on central obesity, dyslipidaemia, hypertension, and glucose intolerance and is associated with an increased risk of cardiovascular diseases and DMT2 as well as all-cause mortality in the general population [10–12]. With PCOS, it has been reported that the prevalence of MetS is approximately 2–3-fold higher among affected women compared to the general population [9, 13]. Women with PCOS also have an increased risk of the individual components of MetS, such as dyslipidaemia [14] and hypertension [15].

Waist circumference (WC) has been commonly used as an index of central obesity. However, WC may be influenced by the time of day, abdominal fullness after meals, respiratory movements, and heavy clothing [16, 17]. In addition, WC may be an inconvenient, inaccurate, and time-consuming measurement in some situations [18, 19]. On the other hand, neck circumference (NC) is a simpler and time-saving anthropometric parameter that reflects subcutaneous adipose tissue of the upper body [20]. Moreover, larger NCs have been found to correlate positively with the individual components of MetS and IR [21, 22]. Interestingly, NC has been suggested to be more strongly associated with cardiometabolic risk factors among women compared to men due to sex differences in free fatty acid metabolism and fat storage [20].

To date, few studies have focused on the use of NC as a metabolic health marker among women with PCOS, although the measure could serve as a simple marker for adverse metabolic outcomes in this population. A study by Liu et al., including 633 women with PCOS, showed that the prevalence of MetS increased significantly from the lowest quartile to the highest quartile of NC [17]. Another study with 121 women found good correlation between NC and MetS among women with PCOS, and the authors suggested replacing WC with NC [23]. Neck circumference was also shown to be a reliable anthropometric measurement to predict risk of IR among women with PCOS [24]. The majority of published studies on NC in PCOS have been small, with control groups lacking and mean NC values varying greatly between them.

Since women with PCOS have a higher risk for multiple abnormalities related to MetS and IR, NC might be a valuable screening tool for estimation of their metabolic risk profile. Early recognition of IR and cardiometabolic risk factors for the prevention of DMT2 and cardiovascular diseases is valuable and cost-efficient [25, 26], also warranting evaluation of the clinical tools available. Therefore, the present systematic review and meta-analysis were conducted to investigate the associations of NC, PCOS and metabolic abnormalities as well as possible cut-off values for NC to recognize MetS and IR among women with PCOS.

## Materials and methods

### Search strategy

The review was conducted through PubMed/Medline and Scopus. The key words used were “neck circumference”, “polycystic ovary syndrome”, “PCOS”, “infertility”, “hirsutism”, “hyperandrogenism”, “testosterone”, “Stein-Leventhal”, “Ferriman-Gallwey”, “FG-score”, “mFG”, “PCO”, “polycystic ovary morphology”, and “PCOM”. We also included the terms “menstrual irregularity”, “menstrual dysfunction”, and “ovarian dysfunction”, however, these terms did not bring out new publications. The search strategy in different databases is shown in Supplementary Appendix 1. The search was performed 28.10.2024. We included only papers written in English and published before 28.10.2024. The full text of the paper needed to be available. We excluded systematic reviews and meta-analyses.

### Study eligibility

We assessed the suitability of the studies by using the population-intervention-control-outcome (PICO) criteria. As a population, we included adult (≥18 years old or referred as adult women in the article) women with PCOS with any form of PCOS diagnosis (Rotterdam criteria, NIH criteria, AE-PCOS criteria, self-reported diagnosis, International Classification of Diseases [ICD] codes). Our intervention was NC measurement. The inclusion criteria regarding definition of control group were not restricted, as the number of studies with controls was limited. Thus, eligible controls could be healthy women without PCOS, women with PCOS from the same study population, or no controls. As an outcome, we included MetS, metabolic risk factors, IR or impaired glucose tolerance, and DMT2. We accepted any form of MetS diagnosis (International Diabetes Federation [IDF] criteria, National Cholesterol Education Program Adult Treatment Panel III [NCEP ATP III] criteria, based on medications or ICD codes), and for the metabolic risk factors we included weight, body mass index (BMI), WC, hip circumference (HC), waist-to-hip-ratio (WHR), hypertension (as reported in the studies), systolic blood pressure (SBP), diastolic blood pressure (DBP), total cholesterol (TC), triglycerides (TG), high density lipoprotein cholesterol (HDL-C), low density lipoprotein cholesterol (LDL-C), fasting plasma glucose (FPG), hemoglobin A1c (HbA1c), fasting insulin (f-INS), homeostasis model assessment of insulin resistance (HOMA-IR), homeostasis model assessment percentage insulin sensitivity (HOMA%S), 2-h result in the oral glucose tolerance test (2 h-OGTT), and high sensitivity C-reactive protein (hs-CRP). For insulin resistance, impaired glucose tolerance, and DMT2, we accepted all diagnostic definitions.

### Data extraction

We were able to identify 139 studies; 71 from PubMed/Medline, and 65 from Scopus. Additionally, three other articles that met the inclusion criteria were found during the review process. The process was documented with systematic review tool by Covidence (Veritas Health Innovation Ltd., Melbourne, Australia). After removing the duplicates (*n* = 25), we had a total of 114 papers for the screening. Two independent reviewers (SMH, NVSK) first screened the papers by title and abstract, excluding 91 studies due to incompatible study design or population, unreported NC measurement, or an outcome that did not fit our PICO definitions. In addition, we excluded articles written in a language other than English. After all exclusions, 23 papers were screened in full text; 13 full texts met the inclusion criteria and were included in the systematic review. From these 13 papers, 8 had a non-PCOS control group and were eligible for the meta-analysis.

### Quality assessment

To assess the quality of the included studies, we used the Newcastle-Ottawa Scale (NOS) for case-control and cohort studies [27, 28], and Jadad Scale for the randomized controlled trials (RCT) [29, 30]. In the case of a cross-sectional study design, we used the adapted form of the Newcastle-Ottawa cohort scale [31–33] (Supplementary Appendix 2). Disagreements were resolved by consensus between reviewers (SMH, NVSK). There are three sections in the NOS: selection of study groups (S), adequacy of adjustment of confounding (C), and ascertainment of the outcome of interest or the exposure (O-E). A study can score a maximum of nine points. If a study was categorized as high-quality meaning low risk of bias, it was defined as fulfilling the pre-determined Selection, Comparability, and Outcome/Exposure criteria: 3–4 points in the Selection (S) domain, 1–2 points in the Comparability (C) domain, and 2–3 points in the Outcome/Exposure (O-E) domain. Studies scoring less in any domain were considered low quality (high risk of bias). Jadad Scale consist of three items: randomization, blinding, and dropout and withdrawals. Total possible score on the Jadad Scale is 5 and studies that scores 3 or more are considered to be of high quality [30]. All the papers included in the study were assessed by two independent reviewers (SMH, NVSK).

### Statistical analysis

A random effects meta-analysis model was conducted to compare NC between PCOS population and non-PCOS controls. The analyses were conducted using R (Version 4.4.1, packages meta and metafor). The Hedges’ *g*, adjusted standardized mean difference (SMD) and its confidence interval (CI) was used as an outcome measure. Heterogeneity was assessed using *Q* statistic, *I*^2^ and *τ*^2^. DerSimonian-Laird estimation method was used for *τ*^2^. A meta-regression was conducted to search the potential sources of heterogeneity. One study [17] was classified as an outlier since its 95% CI (1.43–1.65) did not overlap with the 95% CI of the pooled effect (0.31–1.25) and it was overly influential based on leave-one-out analyses and Baujat diagnostics. The random effects model and the meta-regression were conducted without the outlier study to confirm the original results. Publication bias was evaluated using the funnel plots, rank correlation and regression test for funnel plot asymmetry. *p* values under 0.05 were considered statistically significant and all the *p* values were two-sided.

## Results

**Fig. 1:**
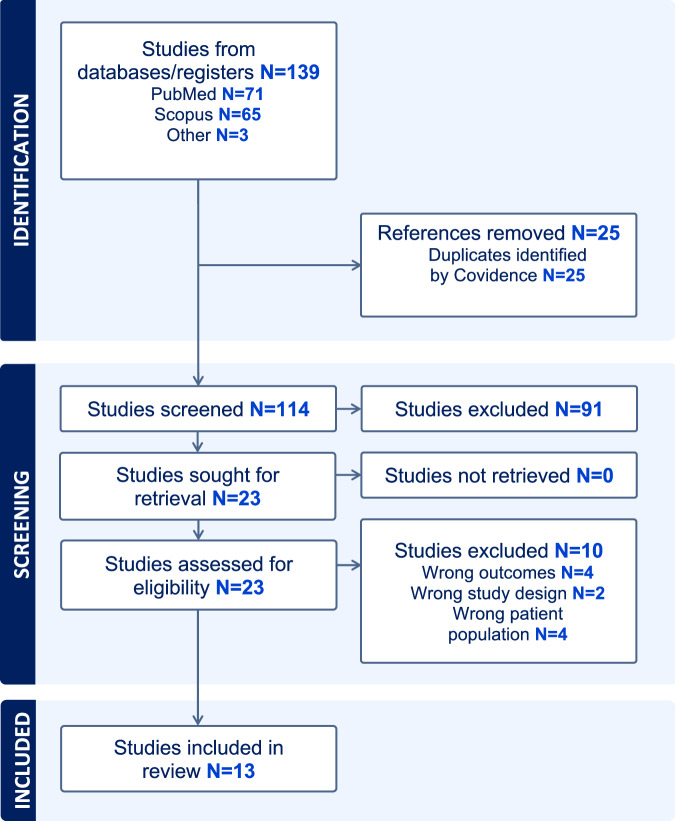
PRISMA flow diagram for the study selection.

The literature search and study selection are presented in the PRISMA flow chart in Fig. 1. Altogether, 13 studies that included eight cross-sectional, four case-control study, and one RCT were included in the review. In total, 3890 women were included: 1469 with PCOS and 2421 without PCOS. The characteristics (study design, PCOS diagnostic criteria, study sample size, ethnicity, mean age, mean NC, assessed outcomes, NOS/Jadad scores) of the included studies are represented in Table 1. Table 2 presents the results of the review categorized according to the main outcomes and shows number of the studies where significantly larger NC was found with significantly higher anthropometric measurements, blood pressure values, lipid and glucose metabolism related values, and prevalence of MetS or IR. The mean ages of the participants varied from 22 to 35 years. Most of the studies included an Asian population (*n* = 9) followed by Middle Eastern (*n* = 1), Australian (*n* = 1), South American (*n* = 1) and North African (*n* = 1). Confounding factors were included in six of the studies [17, 24, 34–37] while the remaining studies reported crude associations. Eight of the studies included a control group of women without PCOS [17, 34–40], while five included only women with PCOS. According to the quality evaluation criteria, six of the studies were categorized as high-quality and seven as low-quality.

**Table 1:** Associations of neck circumference with metabolic abnormalities among women with PCOS.

Study	Study design	PCOS criteria	Participants and ethnicity	Mean age ± SD	Mean NC ± SD	NC p-value between groups	Significant metabolic abnormalities (p<0.05)	Non-significant metabolic abnormalities	NC cut-off values for MetS/IR/obesity	Quality
Chen et al. [24]	Cross-sectional	Rotterdam criteria					Women with PCOS and IR had significantly higher NC, SBP, BMI, WHR, WC, FPG, f-INS, TG, LDL-C, HOMA-IR and lower HDL-C.	No significant group difference in DBP or TC.	NC ≥ 34.25 cm for IR	4/2/3 ^e^
			n = 92 PCOS with IR^a^	26.6 ± 4.7	37.3 ± 3.1	<0.001				High quality
			n = 51 PCOS without IR^a^	28.8 ± 4.2	33.9 ± 2.7					
			Ethnicity: East Asia (China)							
Dixon and O’Brien [34]	Prospective observational cross-sectional	Clinical diagnosis: menstrual irregularity and one of FAI of greater than 5, hirsutism or acne.	n = 30 PCOS	32.0 ± 6.0	42.9 ± 2.7	<0.001	Women with PCOS had significantly higher NC, weight, BMI, WC, HC, f-INS and lower HOMA%S.	No significant group difference in hypertension, diabetes, WHR, or FPG.	NC < 39 cm, low risk^b^.	3/2/2 ^e^
			n = 77 non-PCOS	35.3 ± 6.5	39.7 ± 2.3				NC = 39-42, intermediate risk^b^.	High quality
			Ethnicity: Europe (Australia)						NC > 42 cm, high risk^b^.	
El-Eshmawy et al. [38]	Case control	Rotterdam criteria	n = 40 PCOS	28.7 ± 5.7	37.3 ± 2.0	0.326	Women with PCOS had significantly higher SBP, DBP, FPG, f-INS and HOMA-IR.	No significant group difference in NC, BMI, WC, TC, TG, LDL-C or HDL-C.	NA	1/0/3 ^e^
			n = 40 non-PCOS	29.0 ± 6.7	36.9 ± 1.9					Low quality
			Ethnicity: North Africa (Egypt)							
Inci Coskun et al. [35]	Prospective cross-sectional	Clinically diagnosed	n = 45 PCOS	25.2 ± 5.0	33.6 ± 2.2	<0.001	Women with PCOS had significantly higher NC, BMI, WC, HC, FPG, f-INS, HOMA-IR, TC, TG, LDL-C, and hsCRP.	No significant group difference in HbA1c and HDL-C.	NA	3/1/3 ^e^
			n = 42 non-PCOS	26.6 ± 4.2	31.2 ± 1.5					High quality
			Ethnicity: Asia (Turkey)							
Kamrul-Hasan and Aalpona [41]	Cross-sectional	Rotterdam criteria	n = 200 PCOS	23.3 ± 4.9	34.6 ± 3.0	NA	Women with PCOS and larger NC had significantly higher BMI, WC, SBP, DBP, TG, and prevalence of MetS.	No significant correlation with larger NC and FPG, 2h-OGTT, TC, HDL-C or LDL-C.	NC ≥ 32.75 cm, for abdominal obesity with 87.3% sensitivity and 74.4% specificity.	2/0/3 ^e^
			Ethnicity: South Asia (Bangladesh)							Low quality
									NC ≥ 32.75 cm for overweight/obesity with 88.0% sensitivity and 68.0% specificity.	
									NC ≥ 34.25 cm, for metabolic syndrome (ATP III) with 63.0% sensitivity and 64.0% specificity.	
Koduri et al. [43]	RCT	Rotterdam criteria	Weight loss intervention: n = 4 PCOS baseline	NA	34.4 ± 2.6	0.60		No significant difference in NC, weight, WC, HC or WHR after the intervention compared to baseline.		3 ^f^ High quality
			n = 4 PCOS after 6 months		34.3 ± 2.6					
			Control, usual care: n = 11 PCOS baseline	NA	34.6 ± 1.2	0.28		No significant difference in NC, weight, WC, HC or WHR after usual care compared to baseline.		
			n = 11 PCOS after 6 months		34.6 ± 1.2					
			Ethnicity: South Asia (India)							
Liu et al. [17]	Retrospective cross-sectional	Rotterdam criteria	n = 633 PCOS	Q1 = 28.6 ± 3.7 Q2 = 30.3 ± 3.8 Q3 = 29.5 ± 3.8 Q4 = 30.3 ± 3.5	Q1 = 30.0 ± 1.0 Q2 = 32.5 ± 0.5 Q3 = 34.5 ± 0.5 Q4 = 37.2 ± 1.5	<0.001	Women with PCOS and larger NC had significantly higher BMI, WC, HC, WHR, SBP, DBP, FPG, f-INS, HOMA-IR, TG, LDL-C, lower levels of HDL-C, and higher prevalence MetS^c^.	No significant differences were observed between the quartiles of TC among PCOS women.	Cut-off value NC ≥ 33.00 cm for MetS (IDF) among PCOS women with sensitivity 74.01% and specificity 75.22%.	3/2/3 ^e^ High quality
			n = 2172 non-PCOS	Q1 = 29.4 ± 3.0 Q2 = 29.8 ± 2.8 Q3 = 29.6 ± 2.9 Q4 = 30.2 ± 3.0	Q1 = 29.2 ± 0.9 Q2 = 31.0 Q3 = 32.4 ± 0.5 Q4 = 35.3 ± 1.6	<0.001	Non-PCOS women with larger NC showed elevated BMI, WC, HC, WHR, SBP, and DBP.	No significant differences were observed between the quartiles of FPG, f-INS, HOMA-IR, TC, TG, HDL-C, LDL-C or prevalence of MetS^c^ among non-PCOS women.	Cut-off value NC ≥ 31.00 cm for MetS (IDF) among non PCOS women with sensitivity 82.93% and specificity 61.51%.	
			Ethnicity: Asia (China)							
Malathi et al. [37]	Case-control	Rotterdam criteria	n = 24 PCOS	23.0 ± 4.0	31.8 ± 2.7	0.061	Women with PCOS had significantly higher WC and WHR.	No significant group difference in NC, BMI, weight, SBP, and DBP.	NA	1/2/3 ^e^
			n = 30 non-PCOS	24.2 ± 4.6	30.6 ± 1.9					Low quality
			Ethnicity: South Asia (India)							
Mohapatra & Samantaray [42]	Cross-sectional	Rotterdam criteria	n = 108 PCOS with obesity	28.1 ± 5.7	34.9 ± 2.5	<0.001	Women with PCOS and obesity had significantly higher NC, WHR, HC, WC, weight, f-INS, FPG, and HOMA-IR.	No significant group difference in 2h-OGTT.	NA	4/0/3 ^e^
			n = 35 PCOS without obesity	22.9 ± 5.7	30.1 ± 3.6					Low quality
			Ethnicity: South Asia (India)							
Penaforte et al. [36]	Case-control	Rotterdam criteria	n = 30 PCOS	30.5 ± 5.0	37.8 ± 2.6	0.20	Women with PCOS had significantly higher TGs.	No significant group difference in NC, BMI, and HC.	NA	4/2/3 ^e^
			n = 15 non-PCOS	32.3 ± 5.6	36.8 ± 2.4					High quality
			Ethnicity: South America (Brazil)							
Pillai et al. [23]	Prospective observational cross-sectional	Rotterdam criteria	n = 63 PCOS with MetS (IDF)	26.0 ± 6.0	34.3 ± 2.8	<0.001	Women with PCOS and MetS according to IDF had significantly higher NC, BMI, weight, WC, HC, FPG, f-INS, 2h-OGTT, TG, SBP, DBP, and lower HDL-C compared to women with PCOS but without MetS.	No significant group difference (IDF criteria) in HOMA-IR.	NC ≥ 33.35 cm for MetS (IDF) with sensitivity 60.3% and specificity 70.7%.	4/0/3 ^e^
			n = 58 PCOS without MetS (IDF)	23.5 ± 5.9	32.0 ± 3.7					Low quality
			n = 37 PCOS with MetS (ATP III)	26.1 ± 6.0	35.0 ± 3.2	<0.001	Women with PCOS and MetS according to ATP III had significantly higher NC, BMI, weight, WC, HC, f-INS, TG, SBP, DBP, and HOMA-IR compared to women with PCOS but without MetS.	No significant group difference (ATP III criteria) in FPG, 2h-OGTT, or HDL-C.	NC ≥ 33.87 cm for MetS (ATP III) with sensitivity 73% and specificity 69%.	
			n = 84 PCOS without MetS (ATP III)	24.2 ± 6.1	32.4 ± 3.3					
			Ethnicity: South Asia (India)							
Shirvanizadeh et al. [40]	Observational cross-sectional	Rotterdam criteria	n = 10 PCOS with AO ^d^	28.8 ± 3.3	35.3 ± 1.6	<0.001	Women with PCOS and AO had significantly higher NC and WHR compared to women with PCOS but without AO.	No significant group difference in BMI.	NA	3/0/3 ^e^
			n = 10 PCOS without AO ^d^	28.3 ± 2.3	32.3 ± 1.7					Low quality
			n = 10 non- PCOS with AO ^d^	29.7 ± 3.7	35.0 ± 2.0	<0.001				
			n = 10 non-PCOS without AO ^d^	29.9 ± 4.7	32.3 ± 2.4					
			Ethnicity: Middle East (Iran)							
Shivakumar & Urooj [39]	Case-control	Rotterdam criteria	n = 25 PCOS	23.5 ± 2.2	33.9 ± 3.0	0.013	Women with PCOS had significantly higher NC and weight.	No significant group difference in BMI or HC.	NA	4/0/3 ^e^
			n = 25 non-PCOS	23.2 ± 2.2	31.8 ± 2.7					Low quality
			Ethnicity: South Asia (India)							

^a^Subjects were considered as having IR when the HOMA-IR index was ≥2.6x10^-6^ molxU/L^2^, ^b^Risk for premenopausal women with obesity, ^c^Adjusted Model 3. ^d^ Subjects were considered as having AO when waist/hip ratio was ≥ 0.80. ^e^ Newcastle-Ottawa Scale (S/C/O-E), ^f^ Jadad Scale

AO = abdominal obesity (waist/hip ratio ≥ 0.80), ATP III = National Cholesterol Education Program’s Adult Treatment Panel III report criteria for the metabolic syndrome, BMI = body mass index, DBP = diastolic blood pressure, FAI = free androgen index, FPG = fasting plasma glucose, f-INS = fasting insulin, HbA1c = hemoglobin A1c, HC = hip circumference, HDL-C = high density lipoprotein cholesterol, HOMA-IR = Homeostasis model assessment of insulin resistance, HOMA%S = Homeostasis model assessment percentage insulin sensitivity, IDF = International Diabetes Federation Worldwide Definition of the Metabolic Syndrome, IR = insulin resistance, LDL-C = low density lipoprotein cholesterol, MetS = metabolic syndrome, NA = not applicable, NC = neck circumference, 2h-OGTT = 2 hour oral glucose tolerance test, PCOS = Polycystic ovary syndrome, RCT = randomized controlled trial, SBP = systolic blood pressure, TC = total cholesterol, TG = triglycerides, WC = waist circumference, WHR = waist to hip ratio.

**Table 2:** Associations of neck circumference to metabolic outcomes among women with PCOS.

Outcome	No. of the studies with significantly higher NC and outcome/No. of the studies reporting the outcome	Study	Results: Significantly larger NC and higher outcome in study population	Study population and control groups
BMI	6/11	Dixon and O´Brien [34]	Yes	PCOS vs. non-PCOS
		El-Eshmawy et al. [38]	No	PCOS vs. non-PCOS
		Inci Coskun et al. [35]	Yes	PCOS vs. non-PCOS
		Malathi et al. [37]	No	PCOS vs. non-PCOS
		Penaforte et al. [36]	No	PCOS vs. non-PCOS
		Shivakumar and Urooj [39]	No	PCOS vs. non-PCOS
		Shirvanizadeh et al. [40]	No	PCOS vs. non-PCOS with and without AO
		Kamrul-Hasan and Aalpona [41]	Yes	Women with PCOS according to NC quartiles
		Liu et al. [17]	Yes	Women with PCOS according to NC quartiles
		Chen et al. [24]	Yes	Women with PCOS with IR vs. without IR
		Pillai et al. [23]	Yes	Women with PCOS with MetS vs. without MetS (IDF)
			Yes	Women with PCOS with MetS vs. without MetS (ATP III)
WC	7/10	Dixon and O´Brien [34]	Yes	PCOS vs. non-PCOS
		El-Eshmawy et al. [38]	No	PCOS vs. non-PCOS
		Inci Coskun et al. [35]	Yes	PCOS vs. non-PCOS
		Malathi et al. [37]	No	PCOS vs. non-PCOS
		Kamrul-Hasan and Aalpona [41]	Yes	Women with PCOS according to NC quartiles
		Liu et al. [17]	Yes	Women with PCOS according to NC quartiles
		Chen et al. [24]	Yes	Women with PCOS with IR vs. without IR
		Pillai et al. [23]	Yes	Women with PCOS with MetS vs. without MetS (IDF)
			Yes	Women with PCOS with MetS vs. without MetS (ATP III)
		Koduri et al. [43]	No	Women with PCOS with vs. without weight loss intervention
		Mohapatra and Samantaray [42]	Yes	PCOS with obesity vs. PCOS without obesity
HC	5/8	Dixon and O´Brien [34]	Yes	PCOS vs. non-PCOS
		Inci Coskun et al. [35]	Yes	PCOS vs. non-PCOS
		Penaforte et al. [36]	No	PCOS vs. non-PCOS
		Shivakumar and Urooj [39]	No	PCOS vs. non-PCOS
		Liu et al. [17]	Yes	Women with PCOS according to NC quartiles
		Pillai et al. [23]	Yes	Women with PCOS with MetS vs. without MetS (IDF)
			Yes	Women with PCOS with MetS vs. without MetS (ATP III)’
		Koduri et al. [43]	No	Women with PCOS with vs. without weight loss intervention
		Mohapatra and Samantaray [42]	Yes	PCOS with obesity vs. PCOS without obesity
TG	5/7	El-Eshmawy et al. [38]	No	PCOS vs. non-PCOS
		Inci Coskun et al. [35]	Yes	PCOS vs. non-PCOS
		Penaforte et al. [36]	No	PCOS vs. non-PCOS
		Kamrul-Hasan and Aalpona [41]	Yes	Women with PCOS according to NC quartiles
		Liu et al. [17]	Yes	Women with PCOS according to NC quartiles
		Chen et al. [24]	Yes	Women with PCOS with IR vs. without IR
		Pillai et al. [23]	Yes	Women with PCOS with MetS vs. without MetS (IDF)
			Yes	Women with PCOS with MetS vs. without MetS (ATP III)
LDL-C	3/5	El-Eshmawy et al. [38]	No	PCOS vs. non-PCOS
		Inci Coskun et al. [35]	Yes	PCOS vs. non-PCOS
		Kamrul-Hasan and Aalpona [41]	No	Women with PCOS according to NC quartiles
		Liu et al. [17]	Yes	Women with PCOS according to NC quartiles
		Chen et al. [24]	Yes	Women with PCOS with IR vs. without IR
HDL-C^a^	3/6	El-Eshmawy et al. [38]	No	PCOS vs. non-PCOS
		Inci Coskun et al. [35]	No	PCOS vs. non-PCOS
		Kamrul-Hasan and Aalpona [41]	No	Women with PCOS according to NC quartiles
		Liu et al. [17]	Yes^a^	Women with PCOS according to NC quartiles
		Chen et al. [24]	Yes^a^	Women with PCOS with IR vs. without IR
		Pillai et al. [23]	Yes^a^	Women with PCOS with MetS vs. without MetS (IDF)
			No	Women with PCOS with MetS vs. without MetS (ATP III)
SBP	4/6	El-Eshmawy et al. [38]	No	PCOS vs. non-PCOS
		Malathi et al. [37]	No	PCOS vs. non-PCOS
		Kamrul-Hasan and Aalpona [41]	Yes	Women with PCOS according to NC quartiles
		Liu et al. [17]	Yes	Women with PCOS according to NC quartiles
		Chen et al. [24]	Yes	Women with PCOS with IR vs. without IR
		Pillai et al. [23]	Yes	Women with PCOS with MetS vs. without MetS (IDF)
			Yes	Women with PCOS with MetS vs. without MetS (ATP III)
DBP	3/6	El-Eshmawy et al. [38]	No	PCOS vs. non-PCOS
		Malathi et al. [37]	No	PCOS vs. non-PCOS
		Kamrul-Hasan and Aalpona [41]	Yes	Women with PCOS according to NC quartiles
		Liu et al. [17]	Yes	Women with PCOS according to NC quartiles
		Chen et al. [24]	No	Women with PCOS with IR vs. without IR
		Pillai et al. [23]	Yes	Women with PCOS with MetS vs. without MetS (IDF)
			Yes	Women with PCOS with MetS vs. without MetS (ATP III)
FPG	5/8	Dixon and O´Brien [34]	No	PCOS vs. non-PCOS
		El-Eshmawy et al. [38]	No	PCOS vs. non-PCOS
		Inci Coskun et al. [35]	Yes	PCOS vs. non-PCOS
		Kamrul-Hasan and Aalpona [41]	No	Women with PCOS according to NC quartiles
		Liu et al. [17]	Yes	Women with PCOS according to NC quartiles
		Chen et al. [24]	Yes	Women with PCOS with IR vs. without IR
		Pillai et al. [23]	Yes	Women with PCOS with MetS vs. without MetS (IDF)
			No	Women with PCOS with MetS vs. without MetS (ATP III)
		Mohapatra and Samantaray [42]	Yes	PCOS with obesity vs. PCOS without obesity
f-INS	6/7	Dixon and O´Brien [34]	Yes	PCOS vs. non-PCOS
		El-Eshmawy et al. [38]	No	PCOS vs. non-PCOS
		Inci Coskun et al. [35]	Yes	PCOS vs. non-PCOS
		Liu et al. [17]	Yes	Women with PCOS according to NC quartiles
		Chen et al. [24]	Yes	Women with PCOS with IR vs. without IR
		Pillai et al. [23]	Yes	Women with PCOS with MetS vs. without MetS (IDF)
			Yes	Women with PCOS with MetS vs. without MetS (ATP III)
		Mohapatra and Samantaray [42]	Yes	PCOS with obesity vs. PCOS without obesity
HOMA-IR or HOMA%S^b^	6/7	Dixon and O´Brien [34]	Yes^b^	PCOS vs. non-PCOS
		El-Eshmawy et al. [38]	No	PCOS vs. non-PCOS
		Inci Coskun et al. [35]	Yes	PCOS vs. non-PCOS
		Liu et al. [17]	Yes	Women with PCOS according to NC quartiles
		Chen et al. [24]	Yes	Women with PCOS with IR vs. without IR
		Pillai et al. [23]	Yes	Women with PCOS with MetS vs. without MetS (IDF)
			Yes	Women with PCOS with MetS vs. without MetS (ATP III)
		Mohapatra and Samantaray [42]	Yes	PCOS with obesity vs. PCOS without obesity
Prevalence of MetS or IR	3/3	Kamrul-Hasan and Aalpona [41]	Yes (MetS)	Women with PCOS according to NC quartiles
		Liu et al. [17]	Yes (MetS)	Women with PCOS according to NC quartiles
		Chen et al. [24]	Yes (IR)	Women with PCOS with IR vs. without IR

*AO* abdominal obesity (waist/hip ratio ≥0.80), *ATP III* National Cholesterol Education Program’s Adult Treatment Panel III report criteria for the metabolic syndrome, *BMI* body mass index, *DBP* diastolic blood pressure, *FPG* fasting plasma glucose, *f-INS* fasting insulin, *HC* hip circumference, *HDL-C* high density lipoprotein cholesterol, *HOMA-IR* homeostasis model assessment of insulin resistance, *HOMA%S* homeostasis model assessment percentage insulin sensitivity, *IDF* International Diabetes Federation Worldwide Definition of the Metabolic Syndrome, *IR* insulin resistance, *LDL-C* low density lipoprotein cholesterol, *MetS* metabolic syndrome, *NC* neck circumference, *PCOS* polycystic ovary syndrome, *SBP* systolic blood pressure, *TG* triglycerides, *WC* waist circumference.

^a^Lower HDL-C.

^b^Lower HOMA%S.

### Associations between NC and anthropometric measurements

Seven [17, 23, 24, 34, 35, 41, 42] out of ten studies reported that women with PCOS and larger NC had a significantly larger WC compared to women with smaller NC with or without PCOS. Five studies [17, 23, 34, 35, 42] reported corresponding results for HC and six for BMI [17, 23, 24, 34, 35, 41]. The study by El-Eshmawy et al. [38] compared women with obesity with or without PCOS and found no differences between groups concerning NC, BMI, and WC. Penaforte et al. [36] and Malathi et al. [37] reported similar results. They found no difference in NC, BMI, weight and HC between women with PCOS and non-PCOS controls. Higher WC and WHR was found among women with PCOS in the study by Malathi et al. [37] compared to non-PCOS controls despite no difference in NC. Koduri et al. [43] performed a weight loss intervention study among women with PCOS and found no difference in NC, weight, WC, HC or WHR after the intervention compared to PCOS control group. Four studies [17, 24, 40, 42] found a correlation between higher WHR and higher NC among women with PCOS. Liu et al. [17] and Shirvanizadeh et al. [40] found a corresponding correlation among women without PCOS. However, a correlation between WHR and higher NC was not observed by Dixon and O’Brien [34], who compared women with obesity with or without PCOS. Four studies [23, 34, 39, 42] found that women with PCOS and larger NC had a higher weight when compared to women with or without PCOS. In the study by Inci Coskun et al. [35] women with PCOS had higher NC, fat ratio (%), fat mass, and lean mass when compared to healthy women. Shirvanizadeh et al. [40] concluded that abdominal obesity among women with or without PCOS was associated to larger NC. In addition, higher NC among women with PCOS was seen in the study by Shivakumar and Urooj [39] compared to healthy controls despite no difference in BMI or HC.

### Associations between NC and glucose metabolism parameters

Eight studies reported at least one of the following glucose metabolism parameters: FPG, f-INS, HOMA-IR, HOMA%S, HbA1c, 2h-OGTT or prevalence of diabetes. All eight studies measured FPG, and five studies [17, 23, 24, 35, 42] found larger NC and higher FPG values among women with PCOS. In study by Pillai et al. [23], women with PCOS and MetS according to IDF criteria had higher NC and higher FPG compared to women with PCOS and without MetS. However, when the MetS criteria was changed to ATP III, no significant difference was found in FPG values despite the higher NC. In addition, in the studies by Dixon and O´Brien [34] and Kamrul-Hasan and Aalpona [41], no association was found between higher NC and FPG.

Fasting insulin values were reported in seven of the studies. Larger NC and higher f-INS values were found in six of these studies [17, 23, 24, 34, 35, 42]. Of the seven studies that reported HOMA-IR or HOMA%S values, larger NC and higher HOMA-IR or lower HOMA%S values were found in six [17, 23, 24, 34, 35, 42]. However, in the study by Pillai et al. [23], significantly larger NC and higher HOMA-IR values were seen only among women with PCOS and MetS according to ATP III criteria but not according to IDF criteria. Mohapatra and Samantaray [42] compared women with PCOS and obesity to lean women with PCOS. They found an association with higher NC, FPG, f-INS among women with PCOS and obesity but observed no difference in 2h-OGTT glucose values between groups. Kamrul-Hasan and Aalpona found similar results and reported lack of significant correlation with larger NC and 2h-OGTT values [41].

### Associations between NC and dyslipidemia

Five [17, 23, 24, 35, 41] of the seven studies reported women with PCOS and with larger NC having higher values of TC, LDL-C, or TG, or lower HDL-C. Higher TG concentrations and larger NC were reported in five [17, 23, 24, 35, 41] out of seven studies. Five studies reported serum LDL-C concentrations, and three studies [17, 24, 35] showed higher LDL-C with larger NC. On the other hand, Kamrul-Hasan and Aalpona [41] did not found a correlation between larger NC and LDL-C, and El-Eshmawy et al. [38] found no difference in NC and LDL-C values between women with or without PCOS. Of six studies reporting serum levels of HDL-C, three [17, 23, 24] reported lower HDL-C values with larger NC. Liu et al. [17] divided women with PCOS into quartiles according to NC and found that larger NC was associated with higher TG and LDL-C and lower levels of HDL-C, but no association was found with larger NC and TC. They also examined association with higher NC and lipid values (TC, TG, HDL-C, LDL-C) among women without PCOS and found no significant associations. In addition, Penaforte et al. [36] observed higher TGs among women with PCOS compared to healthy controls, but NC values were not significantly different between groups.

### Associations between NC and blood pressure

Six studies [17, 23, 24, 37, 38, 41] reported SBP and DBP values. Four of them [17, 23, 24, 41] found significantly higher SBP values among women with PCOS and larger NC, and three [17, 23, 41] reported the same for DBP values. In addition, higher SBP, DBP, and NC values were observed among women without PCOS in the study by Liu et al. [17]. El-Eshmawy et al. [38] reported higher SBP and DBP among women with PCOS but found no difference in NC values when compared to women without PCOS. Dixon and O’Brien [34] did not detect a difference between women with PCOS and without PCOS in the prevalence of hypertension even though women with PCOS had a significantly higher NC.

### Use of neck circumference in identifying of metabolic syndrome or insulin resistance

Five studies [17, 23, 24, 34, 41] examined the association of MetS or IR among women with PCOS. Indications of the association with larger NC and MetS or IR were observed in these studies. Larger NC associated significantly with IR after adjusting for confounding factors in the study by Chen et al. [24]. Their study demonstrated that the best cut-off value for recognizing IR among Chinese women with PCOS was ≥34.25 cm.

According to Dixon and O´Brien [34], who studied women with obesity with or without PCOS in an Australian population, of all tested anthropometric measures (NC, WC, HC, WHR, BMI, weight), NC provided the highest significant correlations with hyperinsulinemia, IR and beta cell function. They concluded that with NC of <39 cm the risk for IR was low, between 39 cm and 42 cm intermediate, and over 42 cm high. Women with PCOS had a significantly larger NC compared to women without PCOS in their study.

Kamrul-Hasan and Aalpona [41] examined the association of NC with MetS among South Asian women with PCOS. They found a significantly higher prevalence of MetS with larger NC and determined that the best NC cut-off value for MetS (ATP III) was ≥34.25 cm. Liu et al. [17] reported that NC was strongly and independently associated with MetS among Chinese women with PCOS compared to women without PCOS. The prevalence of MetS increased significantly among women with PCOS from the lowest quartile to the highest quartile of NC. The prevalence ratio for MetS was 9.94 higher in the highest quartile of NC compared to the lowest in the adjusted model. They found that the optimal NC cut-off point for predicting MetS was 33.0 cm among women with PCOS.

Pillai et al. [23] reported similar cut-off values for the prediction of MetS. They studied South Asian women with PCOS and reported that NC value ≥33.35 cm was optimal for predicting MetS according to IDF criteria and ≥33.87 cm according to ATP III criteria. In addition, NC was found to be significantly larger among women with PCOS and MetS compared to women with PCOS but without MetS.

### Meta-analysis of neck circumference between PCOS and controls

**Fig. 2:**
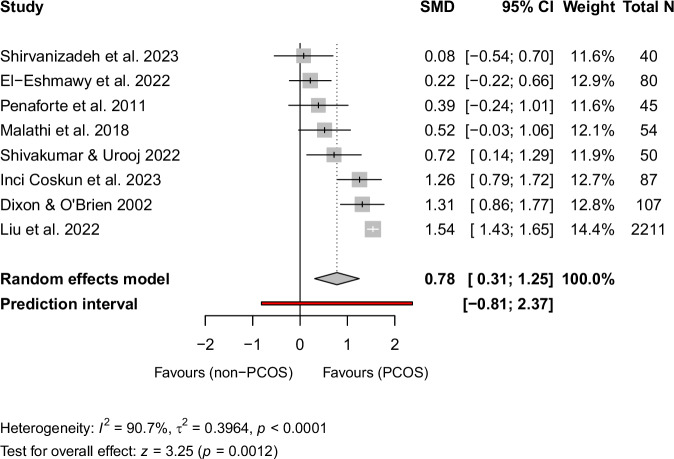
The forest plot comparing NC between women with PCOS and non-PCOS controls.

A total of eight studies were included in the meta-analysis comparing NC between 658 women with PCOS and 2016 without PCOS [17, 34–40]. Quartile 2 in the study by Liu et al. [17], including 189 women with PCOS and 405 non-PCOS controls, was exclude from the meta-analysis since they did not report standard deviation values for the mean NC. The observed SMD ranged from 0.08–1.54. The pooled SMD based on the random effects model was 0.78 (95% CI: 0.31–1.25, *z* = 3.25, *p* = 0.0012) which is very close to large effect size. These results indicate significantly larger NC values among women with PCOS compared to healthy controls without PCOS (Fig. 2). Heterogeneity of the studies was substantial (*I*^2^ = 90.7%, *τ*^2^ = 0.3964, *p* < 0.0001). The study by Liu et al. [17] was an outlier according to the confidence interval analysis (SMD 1.54, 95% CI: 1.43–1.65) which could result from the considerable larger study population compared to other studies. The random effects model was performed without Liu et al. [17] and the pooled SMD remained significant (SMD 0.66, 95% CI: 0.28–1.04, *z* = 3.38, *p* = 0.0007). Heterogeneity of the studies was attenuated after removal of the outlier study but remained substantial (*I*^2^ = 73.2%, *τ*^2^ = 0.1928, *p* = 0.0010).

The sources of heterogeneity were analyzed with the meta-regression (Table 3). Covariates were ethnicity, BMI, BMI among women with PCOS vs. women without PCOS, age, total number of study participants and publication year. These covariates can influence NC based on previous research [17, 44–46] and our review. Asian ethnicity was used as a reference ethnicity since majority of the studies were Asian populations. Significant sources of heterogeneity were ethnicity, BMI difference between women with PCOS and healthy controls, and total number of study participants. The meta-regression for ethnicity revealed that it had a significant contribution to heterogeneity: Middle East and North African differed significantly from Asia (*p* = 0.02). The meta-regression was performed omitting the outlier study by Liu et al. [17] but the results remained similar (Supplementary Table 1).

**Table 3:** Meta-regression analysis.

Covariate	Studies	Coefficients	Lower bound	Upper bound	Std. error	*p* value
BMI	8	0.004	−0.05	0.06	0.03	0.88
BMI PCOS vs. non-PCOS	8	0.19	0.03	0.34	0.08	0.017
Total *n*	8	0.0004	0.00	0.001	0.0002	0.05
Age	8	0.02	−0.10	0.15	0.06	0.73
Publication year	8	−0.015	−0.07	0.04	0.03	0.63
Ethnicity, overall^a^	8					0.0058
Ethnicity, Europe^a^	8	0.24	−0.73	1.21	0.49	0.63
Ethnicity, South America^a^	8	−0.69	−1.75	0.37	0.54	0.20
Ethnicity, Middle East and North Africa^a,b^	8	−0.92	−1.69	0.15	0.39	0.02

^a^Asian ethnicity, including East and South Asia, was used as a reference ethnicity.

^b^Middle East and North Africa was combined to one group.

The publication bias was evaluated using a visual assessment of the funnel plot (Supplementary Fig. 1) and tested with a rank correlation as well as a regression test for funnel plot asymmetry. According to the rank collection there was no publication bias (*p* = 0.1789). However, the regression test showed significant asymmetry (*p* = 0.0050). When the outlier study by Liu et al. [17] was omitted, funnel plot asymmetry was no longer significant (rank correlation test *p* = 0.5619, regression test *p* = 0.1849). This clearly suggest that the study by Liu et al. [17] is a significant source of heterogeneity due to the substantially larger study population compared to other studies.

## Discussion

The current review showed that larger NC was associated with MetS and IR as well as individual components of MetS among women with PCOS. According to the meta-analysis NC was significantly larger among women with PCOS compared to healthy controls (*p* = 0.0012). Moreover, studies reported positive association with larger NC and higher WC, HC, TG, SBP, f-INS, and HOMA-IR or lower HOMA%S and higher prevalence of MetS or IR among women with PCOS. Some of the included studies, but not all, found an association with larger NC and higher BMI, FPG, LDL-C, and DBP, or lower HDL-C among women with PCOS.

### Neck circumference as a substitute for waist circumference

Higher WC has been linked to many chronic diseases and higher mortality [47, 48]. However, WC has some limitations: the correct measuring site may be challenging to find, the measurement can easily vary between two measurements, abdominal fullness and state of the expiration affect the results, and it can be unpleasant for the patient [16]. Also, self-measurement may be more difficult compared to NC measure. Measurement of NC is not affected by similar problems and has been found to correlate well with the other anthropometric measurements in the general population [49]. Based on our systematic review, this also applied to women with PCOS. We found that larger NC had a high correlation with WC and HC in PCOS compared to controls in majority of the included studies. However, correlation to BMI was not as strong. Some of the studies observed no difference in BMI despite higher NC among PCOS population compared to healthy controls [39, 40]. This indicates that abdominal obesity, a common feature in PCOS [50], has a high correlation to NC regardless BMI. Despite the limitations of WC, it has been proven to be a valid measurement of metabolic abnormalities. Our data indicates that the use of NC and WC do not contradict one another and can be used simultaneously in clinical practice.

### Neck circumference and glucose metabolism

Insulin resistance is strongly linked to PCOS pathogenesis [9]. Hyperinsulinemia is one of the first indications of IR, and dysfunction of insulin clearance is a sign of metabolic health abnormality [51]. Multiple studies have established correlations with higher NC and IR among general population [22, 52, 53]. Study by Yang et al. [53] suggested that NC was more reliable than other anthropometric measurements as a predictor for IR. Jurczewska et al. [54] observed a higher prevalence of IR in PCOS among women with higher visceral adipose tissue which is closely linked to larger NC [45]. In this review, corresponding results were found. We observed significantly higher f-INS, FPG, and HOMA-IR or lower HOMA%S values with higher NC among women with PCOS in most of the included studies. Only one study, by El-Ehsmawy et al. [38], showed contradictory results, but the study population was small and highly selected (only subjects with obesity). We also found a stronger connection with higher f-INS and HOMA-IR values and larger NC compared to FPG. This could be explained by the fact that in an IR state, insulin secretion is enhanced, which results in hyperinsulinemia [55]. Eventually, pancreatic beta cells fail to produce enough insulin for IR compensation leading to a rise in blood glucose levels [56]. Therefore, it has been suggested that hyperinsulinemia exists before the rise of blood glucose levels [56], which could explain the weaker association with larger NC and higher FPG in our review.

### Neck circumference and dyslipidaemia

Dyslipidaemia is a common abnormality among women with PCOS [57]. Low HDL-C, high LDL-C and high TGs are a sign of abnormal lipid metabolism [58]. In this review, we found indications of associations between larger NC and higher LDL-C as well as higher TG levels. The connection was stronger with TG values. High TG levels predict a higher cardiovascular risk [59], and elevated TG levels are highly prevalent in subjects with MetS [60]. In line with our findings, women with PCOS have been found to have higher levels of TGs compared to controls in other studies [36, 61]. In addition, high LDL-C is a well-established risk factor for cardiovascular disease [61]. Studies have demonstrated that women with PCOS have higher LDL-C values compared to controls [61, 62]. Part of the included studies in this review found higher NC together with higher LDL-C values among women with PCOS [17, 24, 35]. Liu et al. [17] demonstrated that women with PCOS and larger NC had a significantly higher LDL-C, but the same association was not found in women without PCOS. This indicates that larger NC is more strongly associated with dyslipidaemia among women with PCOS compared to the general population. We found that lower HDL-C was associated with larger NC among women with PCOS in only half of the studies [17, 23, 24]. The ethnicity of the study populations in our review may explain the lack of association. A meta-analysis by Namazi et al. [63] found a stronger association among larger NC and lower serum levels of HDL-C among European and North American populations compared to Asian societies. The majority of our study groups were Asian, which may affect the results.

### Neck circumference and blood pressure

In the current review, higher SBP values were found with larger NC among women with PCOS in majority of the included studies. Numerous studies have established higher blood pressure values among women with PCOS [15, 64–66]. However, obesity was not controlled in some of these studies, even though obesity itself is a significant risk factor for hypertension [15]. In the study by Joham et al. [67], the incidence rate of hypertension was four times higher among women with PCOS and obesity compared to age-matched lean women with PCOS. In our review, two studies did not find an association between larger NC and higher blood pressure values [34, 38]. However, these studies only included women with obesity, which could interfere with the associations of NC and blood pressure.

### Neck circumference and prevalence of metabolic syndrome and insulin resistance

It is well established that MetS and IR are more common among women with PCOS [3, 9, 13]. Several studies included in this review found an association with higher NC and prevalence of MetS or IR, and no contradictory result were found [17, 23, 24, 34, 41]. According to Kamrul-Hasan and Aalpona [41] the prevalence of MetS was almost 3-fold in the highest quartile of NC compared to lowest among women with PCOS. In the study by Liu et al. [17], the prevalence of MetS among women with PCOS was almost 10-fold in the highest quartile of NC when compared to lowest. In the same study, the corresponding risk in the highest NC quartile among women without PCOS was only 3.23-fold higher. These results strengthen the importance of NC as a clinical marker for MetS or IR especially among women with PCOS.

### Neck circumference cut-off values for women with PCOS

In clinical practice, NC could be a valuable and simple screening tool for detecting high metabolic risk among women with PCOS. Early identification of MetS and IR is important for the prevention of cardiovascular diseases and DMT2. Therefore, NC cut-off values for detecting the risk of MetS and IR are needed. In this review, cut-off values for MetS varied from 33 cm to 34.25 cm among women with PCOS. Liu et al. [17] defined the NC cut-off value for MetS also among women without PCOS as well and found it to be considerably smaller at ≥31 cm. These results indicate that women with PCOS are more prone to abnormal upper body fat accumulation, which has been shown in other studies as well [50, 68–71]. In this review, NC cut-off values for the IR varied from 34.25 cm up to 42 cm for women with PCOS. Dixon and O’Brien [34] found considerably higher NC cut-off values for IR among Australian women than did Chen et al. [24] among Chinese women. Ethnic variation most likely explains the notable difference in cut-off values between the different studies. Several other studies have defined NC cut-off values among the general population for identification of MetS and IR [44, 63, 72, 73]. In these studies, cut-off values for MetS and IR have varied between 33 cm and 37 cm. In general, the NC cut-off points for MetS or IR seems to be smaller among Asian population compared to other ethnic populations, and the results from this review confirm this.

### Neck circumference between PCOS and controls

The evidence from this meta-analysis showed significantly higher NC values in PCOS compared to healthy controls. The result remained significant after removal of one outlier study. However, heterogeneity of the result was considerable (90.7%). According to the meta-regression, overall ethnicity, Middle East and North African ethnicity, BMI difference between women with PCOS and controls, and total number of study subjects were significant sources of heterogeneity.

Most of the included study populations were Asian populations which most likely impacted on NC. The meta-regression confirmed that ethnicity had a significant effect on results. In our study, Asian study population had generally lower NC values ranging from 30 cm to 37 cm compared to other ethnic populations where the corresponding values where 32 cm to 43 cm. This has been seen other studies as well. Ebrahami et al. [44] studied Iranian population where mean NC was 35.52 cm among women. In South American population mean NC for women was 35.7 cm in the study by Diaz et al. [46]. However, in a large Chinese study including 5428 women, mean NC was considerably smaller 31.8 cm [45]. These results are in line with our findings and highlight the need for wider ethnic studies concerning the use of NC.

In addition, the meta-regression showed that BMI difference between PCOS population and controls had a significant impact on study results. Thus, significantly higher NC values seen in PCOS population were most likely affected by BMI difference between groups. Publication bias was seen when all the studies were included in the analysis but attenuated when the outlier study was removed. Indication of the publication bias is probably due to low overall number of studies.

### Limitations

Our review has several strengths. We used a broad range of outcomes, which resulted in wide inclusion of studies. The associations of NC to each outcome were assessed separately to include extensive health aspects. The quality assessment of the included studies was performed. In addition, we completed a meta-analysis including majority of the studies and assessed sources of heterogeneity as well as publication bias. However, the review has a few limitations. First, most studies included in the review used cross-sectional study designs. Therefore, cause and effect relationship between higher NC and outcomes cannot be established. Second, the study populations consisted of mainly Asian women. There are indications that the ethnicity of a population greatly affects NC cut-off values for recognizing MetS or IR. Therefore, our results may not be generalized to the global population. Third, some of the included studies reported only crude results without controlling confounding factors such as BMI. Obesity itself is a risk factor for MetS as well as IR, affects anthropometric measurements, and is common among women with PCOS. Therefore, the results of this review should be interpreted with caution. Fourth, over half of the included studies were of low-quality according to our quality assessment.

## Conclusions

In summary, results demonstrated increased NC among women with PCOS compared to healthy controls. The review indicated that women with PCOS and larger NC were more insulin resistant, had more metabolic syndrome-related abnormalities such as a higher WC, lipid, and blood pressure values when compared to women with smaller NC with or without PCOS. Studies concerning the usefulness of NC among women with PCOS are scarce, but a large number of studies have reported corresponding results among the general population [21, 63, 74, 75]. These studies have linked greater NC to factors of MetS, dyslipidaemia, and increased risk of DMT2. In this review, possible NC cut-off values for higher risk for MetS and IR varied from 33 cm to 42 cm depending on the study population. More high-quality studies are needed especially among various ethnic populations for the definition of NC cut-off values for MetS and IR in PCOS. Neck circumference could be a useful and practical screening tool for IR and MetS among women with PCOS.

## Supplementary information

Supplementary material information

Supplementary Material

## Data Availability

All data generated or analyzed during this study are included in this published article.
