# Supplementary material for: Neck circumference as a metabolic health marker among women with polycystic ovary syndrome (PCOS): a systematic review and meta-analysis
Source: Int J Obes (Lond). 2025 Apr 21;49(6):998–1012. doi: 10.1038/s41366-025-01753-1 (PMC12158769; doi:10.1038/s41366-025-01753-1)
Supplement: Supplementary file 1 — Supplementary material information [file 41366_2025_1753_MOESM1_ESM.pdf]

Supplementary material information – Text summary and Figure/Table Legends

Supplementary Appendix 1

- Supplementary Appendix 1 includes specific search strategy in Pudmed/Medline and Scopus for the systematic search.
- File format pdf.

Supplementary Appendix 2

- Supplementary Appendix 2 represent questions of the adapted Newcastle-Ottawa quality assessment scale for the cross-sectional studies.
- File format pdf.

Supplementary Figure 1. The funnel plot for the publication bias.

- Supplementary Figure 1 includes the funnel plot figure for the publication bias.
- File format pdf.

Supplementary Table 1. Meta-regression analysis without the outlier study by Liu et al.

- Supplementary Table 1 represent the results of the meta-regression without the outlier study.
- File format pdf.

Figure and table legends for supplementary material

Supplementary Figure 1. The funnel plot for the publication bias.

Supplementary Table 1. Meta-regression analysis without the outlier study by Liu et al.
