# Supplementary material for: Neck circumference as a metabolic health marker among women with polycystic ovary syndrome (PCOS): a systematic review and meta-analysis
Source: Int J Obes (Lond). 2025 Apr 21;49(6):998–1012. doi: 10.1038/s41366-025-01753-1 (PMC12158769; doi:10.1038/s41366-025-01753-1)
Supplement: Supplementary file 2 — Supplementary Material [file 41366_2025_1753_MOESM2_ESM.pdf]

Neck circumference as a metabolic health marker among women with polycystic ovary syndrome (PCOS): A systematic review

Search strategy

Pubmed/Medline

((("Neck"[Mesh]) OR ("neck circumference"[Title/Abstract])) AND (("Polycystic Ovary Syndrome"[Mesh]) OR ("Infertility"[Mesh]) OR ("Hirsutism"[Mesh]) OR ("Hyperandrogenism"[Mesh]) OR ("Testosterone"[Mesh]) OR ("pcos"[Title/Abstract] OR "testosterone"[Title/Abstract] OR "infertility"[Title/Abstract] OR "stein-Levent*"[Title/Abstract] OR "Ferriman-GallWey*"[Title/Abstract] OR "FG score"[Title/Abstract] OR "mFG"[Title/Abstract] OR "hirsutism"[Title/Abstract] OR "hyperandrogenism"[Title/Abstract] OR "pco" [Title/Abstract] OR "pcom" [Title/Abstract] OR "polycystic ovary morphology" [Title/Abstract] OR "polycystic ovary syndrome"[Title/Abstract]))) AND (english[Filter])

Scopus

(TITLE-ABS-KEY ("neck circumference") AND TITLE-ABS-KEY (pcos) OR TITLE-ABS-KEY (testosterone) OR TITLE-ABS-KEY (infertility) OR TITLE-ABS-KEY ("stein-levent*") OR TITLE-ABS-KEY ("ferriman-gallwey") OR TITLE-ABS-KEY ("FG score") OR TITLE-ABS-KEY (mfg) OR TITLE-ABS-KEY (hirsutism) OR TITLE-ABS-KEY (hyperandrogenism) OR TITLE-ABS-KEY (pco) OR TITLE-ABS-KEY (pcom) OR TITLE-ABS-KEY ("polycystic ovary morphology") OR TITLE-ABS-KEY ("polycystic ovary syndrome"))

NEWCASTLE - OTTAWA QUALITY ASSESSMENT SCALE (adapted for cross sectional studies)

Selection: (Maximum 4 points)

1) Representativeness of the cases:

a) Truly representative of the average women with PCOS in the community. *

b) Somewhat representative of the average women with PCOS in the community. *

c) Selected group of participants eg. nurses, volunteers.

d) No description of the derivation of the study group

2) Sample size:

a) Justified and satisfactory *

b) Not justified

3) Non-Response rate

a) Comparability between respondents and non-respondents characteristics is established, and the response rate is satisfactory. *

b) The response rate is unsatisfactory, or the comparability between respondents and non- respondents is unsatisfactory, or no description.

4) Ascertainment of the exposure:

a) Validated screening tool. *

b) Non-validated screening tool, but the tool is available or described. *

c) No description of the measurement tool.

Comparability: (Maximum 2 points)

1) The potential confounders were investigated by subgroup analysis or multivariable analysis.

a) The study investigates potential confounders (select the most important factor and any additional factor for extra point) */**

b) The study does not investigate potential confounders.

Outcome: (Maximum 3 points)

1) Assessment of the outcome:

a) Independent blind assessment. **

b) Record linkage. **

c) Self report.

d) No description.

2) Statistical test:

a) The statistical test used to analyze the data is clearly described and appropriate, and the measurement of the association is presented, including confidence intervals and the probability level (p value). *

b) The statistical test is not appropriate, not described or incomplete.

*1 point

**2 points

This scale has been adapted from the Newcastle-Ottawa Quality Assessment Scale to perform a quality assessment of cross-sectional studies for the systematic review, "Neck circumference as a metabolic health marker among women with polycystic ovary syndrome (PCOS): A systematic review and meta-analysis".

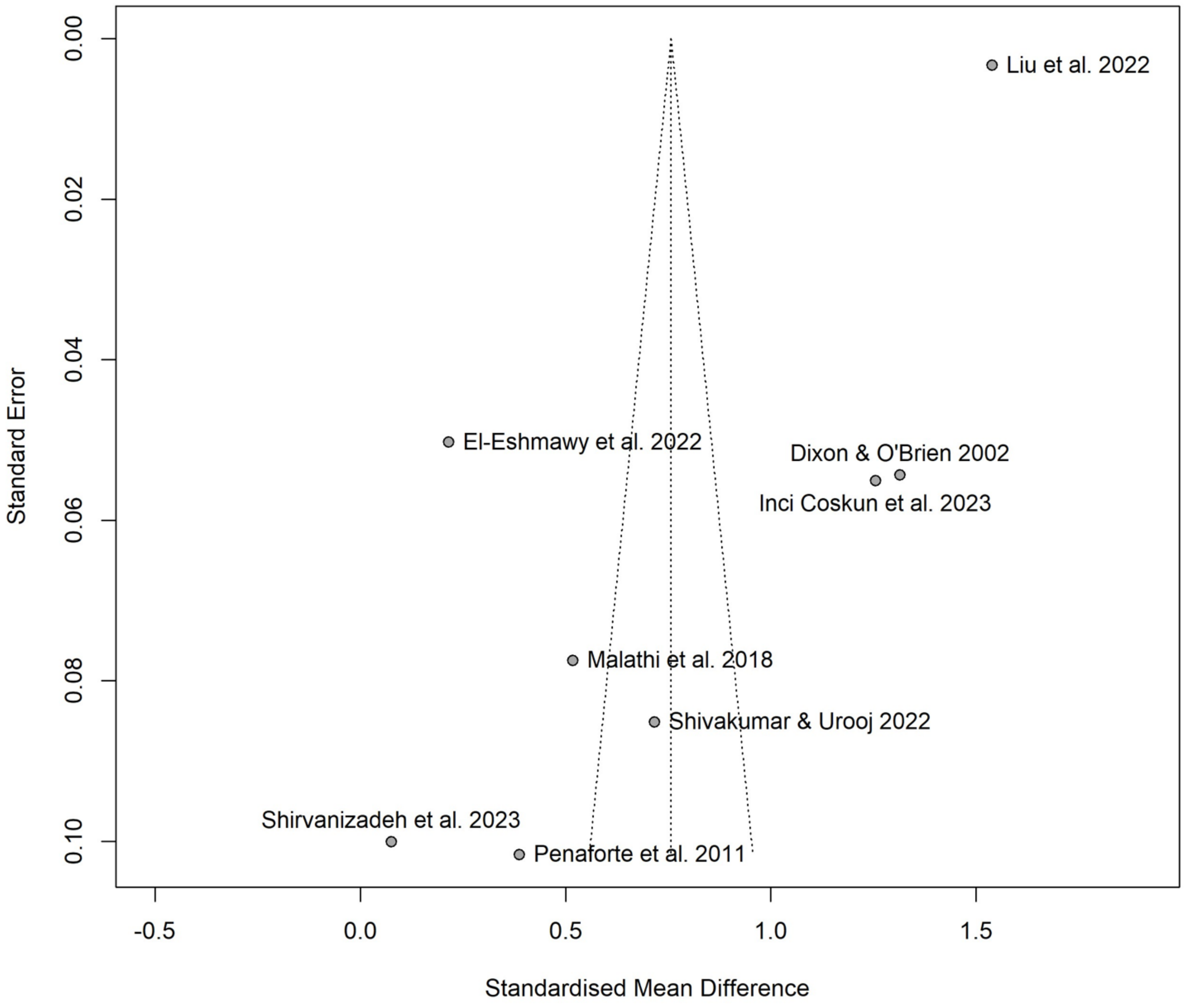

Supplementary Figure 1. The funnel plot for the publication bias.

Supplementary Table 1. Meta-regression analysis without the outlier study by Liu et al.

Covariate	Studies	Coefficients	Lower bound	Upper bound	Std. error	p value
BMI	7	0.019	-0.03	0.06	0.02	0.42
BMI PCOS vs. non-PCOS	7	0.20	0.11	0.29	0.04	<0.0001
Total n	7	0.01	0.002	0.027	0.006	0.02
Age	7	0.02	-0.10	0.15	0.06	0.73
Publication year	7	-0.03	-0.08	0.02	0.02	0.27
Ethnicity, overall[a]	7					0.017
Ethnicity, Europe[a]	7	0.45	-0.27	1.18	0.37	0.22
Ethnicity, South America[a]	7	-0.48	-1.32	0.37	0.43	0.27
Ethnicity, Middle East and North Africa[a,b]	7	-0.70	-1.31	-0.10	0.31	0.02

[a]Asian ethnicity, including East and South Asia, was used as a reference ethnicity, [b]Middle East and North Africa was combined to one group.
